# Epidemiological Survey of *Mycoplasma bovis* in Yaks on the Qinghai Tibetan Plateau, China

**DOI:** 10.1155/2021/6646664

**Published:** 2021-04-14

**Authors:** Jiaqiang Niu, Keiwei Li, Huachun Pan, Xing Gao, Jiakui Li, Dongjing Wang, Mingshuai Yan, Yefen Xu, Suolang Sizhu

**Affiliations:** ^1^Tibet Agriculture and Animal Husbandry College, Linzhi 860000, China; ^2^College of Veterinary Medicine, Huazhong Agricultural University, Wuhan 430070, China

## Abstract

*Mycoplasma bovis* (*M*. *bovis*) is one of the most important pneumonia pathogens in yaks. It may result in more economic losses due to the cold and anoxia condition at Qinghai Tibetan plateau. However, to date, limited information on *M*. *bovis* infection in yaks is available in China. For this purpose, the seroprevalence of *M*. *bovis* was investigated in yaks living in the mentioned area through commercial ELISA kits. A total of 959 yaks were incorporated into this study. The prevalence of the disease in yaks was 48.70%. The serological results revealed a relatively high prevalence of *M*. *bovis* infection in yaks. The present study may greatly contribute to the prevention of this disease. More importance should be given to the potential threat caused by *M*. *bovis* in the special plateau.

## 1. Introduction

Qinghai Tibetan plateau is a particular environment with high altitude, cold, and anoxic conditions [1]. Yak (*Bos grunniens*) is a unique bovine species that is mainly distributed in this plateau. More than 18 million yaks live on the Qinghai-Tibet plateau, whether only a few yaks are found in India, Bhutan, Sikkim, Afghanistan, and Pakistan [2]. This unique species is essential for Tibetans because of the associated milk, wool, and meat production [3].

In 1961, *Mycoplasma bovis* (*M*. *bovis*) was isolated in America and first described as a cause of respiratory disease in 1976 [4]. Due to *M*. *bovis*, infection resulted in high economic losses every year due to pneumonia, mastitis, arthritis, keratoconjunctivitis, otitis, reproductive tract inflammation, abortion, and infertility [5]. In 2008, *M*. *bovis* was first isolated from the lungs of taurine cattle (*Bos taurus*) with pneumonia in China. Since then, it had broken out in some areas of China [6]. The incidence and mortality rates were 50-100% and 10-50%, respectively [7]. With the yak industry's development, large-scale farming is expanding rapidly, which leads to the prevalence of significant infectious diseases on the Qinghai-Tibet plateau. However, to date, limited information on *M*. *bovis* infection in yaks is available on the Qinghai-Tibet plateau [8].

In this study, enzyme-linked immunosorbent assay (ELISA) kits were used to investigate the prevalence of *M*. *bovis* in yaks on the Qinghai-Tibet plateau. The information about the prevalence of *M*. *bovis* on this plateau may be of interest to the livestock owners and relevant departments. It may provide a certain reference for this disease's prevention and control strategies.

## 2. Materials and Methods

### 2.1. Ethics Statement

All procedures were approved and performed by the Laboratory Animals Research Centre of Hubei, Qinghai, Sichuan, Gansu, and Tibet in China and the Ethics Committee of Tibet Agriculture and Animal Husbandry College, China. All animal experiments and procedures were conducted under the relevant guidelines of Proclamation of the Standing Committee of Tibet People's Congress, China.

### 2.2. Serum Samples

**Figure 1:**
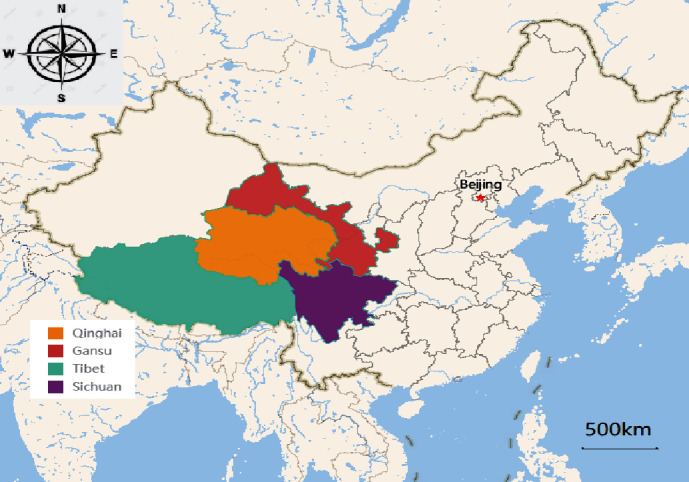
Geographic distribution of the yaks enrolled in the study.

Blood samples from 959 yaks were collected on the Qinghai-Tibet plateau during June 2019 to September 2020 (Qinghai: 224, Gansu: 243, Tibet: 436, and Sichuan: 56) (Figure 1). All the animals that were investigated in this study had no obvious clinical symptoms. Samples were collected mainly from the highland grazing areas, where owners have not recorded obvious symptoms in their herds. Also, herders did not take any protective measures to prevent and cure diseases. The detail information related to the region, gender, and age was reported on prescribed proforma. All the blood samples were centrifuged at 1000 rpm for 10 min, and then, serum was separated and stored at −20°C until further analysis.

### 2.3. Determination of Antibodies against *M*. *bovis*

All serum samples were tested for anti-*M*. *bovis* according to the manufacturer's instructions, bovis antibodies by using two commercial ELISA kits (one was from IDEXX Laboratories, Inc, Beijing, China; the other one came from Shanghai Enzyme-linked Biotechnology Co., Ltd., Shanghai, China). The test value was based on the optical density values at 450 nm (OD 450). To ensure validity, the average OD 450 of positive controls was ≥1.00; the average OD 450 of negative controls was ≤0.15. The positive and negative controls were endowed in the kit and included in each test. The cutoff value was equal to the average OD 450 of negative controls plus 0.15. The results were considered positive when the OD 450 was higher or equal to the cutoff value and considered negative when the OD 450 was lower than the cutoff value. It was judged to be positive when both results were positive and vice versa. In the case of only one positive out of two, IDEXX was used for reexamination.

### 2.4. Statistical Analysis

A multivariable logistic regression model (MLRM) was used to assess significant variables (region, gender, and age) by using the IBM SPSS Statistics 25.0 (SPSS Somers, NY). Statistically significant levels within factors and interactions were accepted when the probability *p* value was found ≤0.05. Odds ratios (OR) was kept at 95% confidence intervals (CI).

## 3. Results

A total of 467/959 (48.70%) yaks were reported positive for *M*. *bovis*. In Qinghai, Gansu, Tibet, and Sichuan, the prevalence was 45.09%, 53.09%, 46.10%, and 64.29%, respectively. Regarding gender, the prevalence in male and female was 45.71% and 50.24%, respectively. If we consider the yaks' age, the prevalence ranged from 45.23% to 55.70% (Table 1).

**Figure 2:**
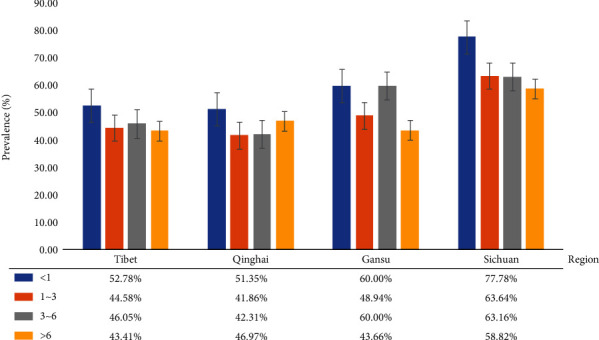
Prevalence of *M*. *bovis* infection at different age.

**Figure 3:**
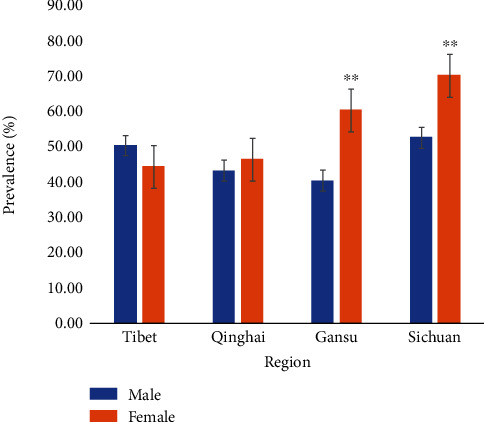
Prevalence of *M*. *bovis* infection in male and female yaks.

According to conditional stepwise logistic regression, region, gender, and age were assessed as potential risk factors in the present study. Furthermore, Sichuan's yaks had a 2.19 times higher risk of infection than yaks in Qinghai (*p* = 0.01) (Table 1). Yaks in Gansu showed 1.38 times higher risk of infection than yaks in Qinghai (Table 1). Meanwhile, yaks having <1 year age had a 1.52 times higher risk of infection as compared to yaks in 3 ≤ years < 6 (*p* < 0.05) (Table 1 and Figure 2), while no significant difference was found in male and female yaks (Table 1 and Figure 3).

## 4. Discussion

The bovine industry has experienced many outbreaks caused by *M*. *bovis* worldwide [9]. Previous reports showed that more than 30% of pneumonia was caused by *M*. *bovis* in Europe, and the incidence rate was high (70%) in America [10]. At present, *M*. *bovis* has distributed worldwide; it also had reported in many regions of China. In our study, the overall prevalence of *M*. *bovis* in yaks was 48.70%. Similar levels of *M*. *bovis* infection were documented in Shanghai (42.1%) [11]. *M*. *bovis* infection prevalence was less than taurine cattle in the Xinjiang Uyghur Autonomous Region (86.3%) [12].

In different regions, the prevalence of *M*. *bovis* in Sichuan was higher than in Tibet, Gansu, and Qinghai. The highest prevalence was detected in calf because of the weak resistance, strong stress response, and vertical transmission [13]. In general, the prevalence of *M*. *bovis* was no significant difference in gender. But in Gansu and Sichuan, the prevalence of *M*. *bovis* in females was higher than that in males which might be due to the weak resistance, especially during pregnancy and parturition. These findings agree with the report by Xie et al. [14].

With the yak industry's rapid development, the high infection of *M*. *bovis* in yaks might be due to the frequent trade and lack of strict quarantine. In addition, drug resistance is an increasing problem worldwide, and there are few commercial vaccines for the prevention of *M*. *bovis* [15].

In conclusion, this study described a high prevalence rate of *M*. *bovis* infection in yaks on the Qinghai-Tibet plateau. Therefore, more attention should be paid to assess the impact of *M*. *bovis* infection fully. Effective measures should also be taken to control the spread of *M*. *bovis* by considering the role of various factors [16].

## Figures and Tables

**Table 1:** Prevalence and risk factors of *M*. *bovis* infection in yaks on the Qinghai Tibetan plateau.

Variable	Category	Number of samples	Positive samples	% (95% CI)	*p* value	OR (95% CI)
Region	Qinghai	224	101	45.09 (38.45-51.86)	Reference	
	Gansu	243	129	53.09 (46.60-59.50)	0.08	1.38 (0.96-1.98)
	Tibet	436	201	46.10 (41.35-50.91)	0.81	1.04 (0.75-1.44)
	Sichuan	56	36	64.29 (50.36-76.64)	0.01	2.19 (1.20-4.02)

Gender	Male	326	149	45.71 (40.21-51.28)	Reference	
	Female	633	318	50.24 (46.27-54.20)	0.18	1.20 (0.92-1.57)

Age	0 < year < 1	158	88	55.70 (47.59-63.58)	0.04	1.52 (1.03-2.25)
	1 ≤ year < 3	184	85	46.20 (38.83-53.68)	0.84	1.04 (0.72-1.51)
	3 ≤ year < 6	283	128	45.23 (39.33-51.23)	Reference	
	Year > 6	334	166	49.70 (44.21-55.19)	0.27	1.20 (0.87-1.64)

Total		959	467	48.70 (45.49-51.91)		

## Data Availability

All the data in the current study could be available by contacting the corresponding author.
